# Complexities underlying the breeding and deployment of Dutch elm disease resistant elms

**DOI:** 10.1007/s11056-021-09865-y

**Published:** 2021-07-28

**Authors:** Juan A. Martín, Jorge Domínguez, Alejandro Solla, Clive M. Brasier, Joan F. Webber, Alberto Santini, Clara Martínez-Arias, Louis Bernier, Luis Gil

**Affiliations:** 1 Departamento de Sistemas y Recursos Naturales, ETSI Montes, Forestal y del Medio Natural, Universidad Politécnica de Madrid, Ciudad Universitaria s/n, 28040 Madrid, Spain; 2 Centro Nacional de Recursos Genéticos Forestales Puerta de Hierro. TRAGSA., Ctra. de la Coruña, Km 7.5, 28040 Madrid, Spain; 3 Faculty of Forestry, Institute for Dehesa Research (INDEHESA), University of Extremadura, Avenida Virgen del Puerto 2, 10600 Plasencia, Spain; 4 Forest Research, Alice Holt Lodge, Farnham, GU10 4LH UK; 5 Istituto per la Protezione Sostenibile delle Piante – C.N.R., Via Madonna del Piano, 10, 50019 Sesto Fiorentino, Italy; 6 Centre d’étude de la Forêt (CEF), Université Laval, Quebec City, QC G1V 0A6 Canada; 7 Southern Swedish Forest Research Centre, Swedish University of Agricultural Sciences (SLU), Alnarp, Sweden

**Keywords:** Dutch elm disease, Breeding, Resistance, *Ulmus*, *Ophiostoma ulmi*, *Ophiostoma novo-ulmi*, Phenotypic plasticity

## Abstract

Dutch elm disease (DED) is a vascular wilt disease caused by the pathogens *Ophiostoma ulmi* and *Ophiostoma novo-ulmi* with multiple ecological phases including pathogenic (xylem), saprotrophic (bark) and vector (beetle flight and beetle feeding wound) phases. Due to the two DED pandemics during the twentieth century the use of elms in landscape and forest restoration has declined significantly. However new initiatives for elm breeding and restoration are now underway in Europe and North America. Here we discuss complexities in the DED ‘system’ that can lead to unintended consequences during elm breeding and some of the wider options for obtaining durability or ‘field resistance’ in released material, including (1) the phenotypic plasticity of disease levels in resistant cultivars infected by *O. novo-ulmi*; (2) shortcomings in test methods when selecting for resistance; (3) the implications of rapid evolutionary changes in current *O. novo-ulmi* populations for the choice of pathogen inoculum when screening; (4) the possibility of using active resistance to the pathogen in the beetle feeding wound, and low attractiveness of elm cultivars to feeding beetles, in addition to resistance in the xylem; (5) the risk that genes from susceptible and exotic elms be introgressed into resistant cultivars; (6) risks posed by unintentional changes in the host microbiome; and (7) the biosecurity risks posed by resistant elm deployment. In addition, attention needs to be paid to the disease pressures within which resistant elms will be released. In the future, biotechnology may further enhance our understanding of the various resistance processes in elms and our potential to deploy trees with highly durable resistance in elm restoration. Hopefully the different elm resistance processes will prove to be largely under durable, additive, multigenic control. Elm breeding programmes cannot afford to get into the host–pathogen arms races that characterise some agricultural host–pathogen systems.

## Introduction

**Fig. 1:**
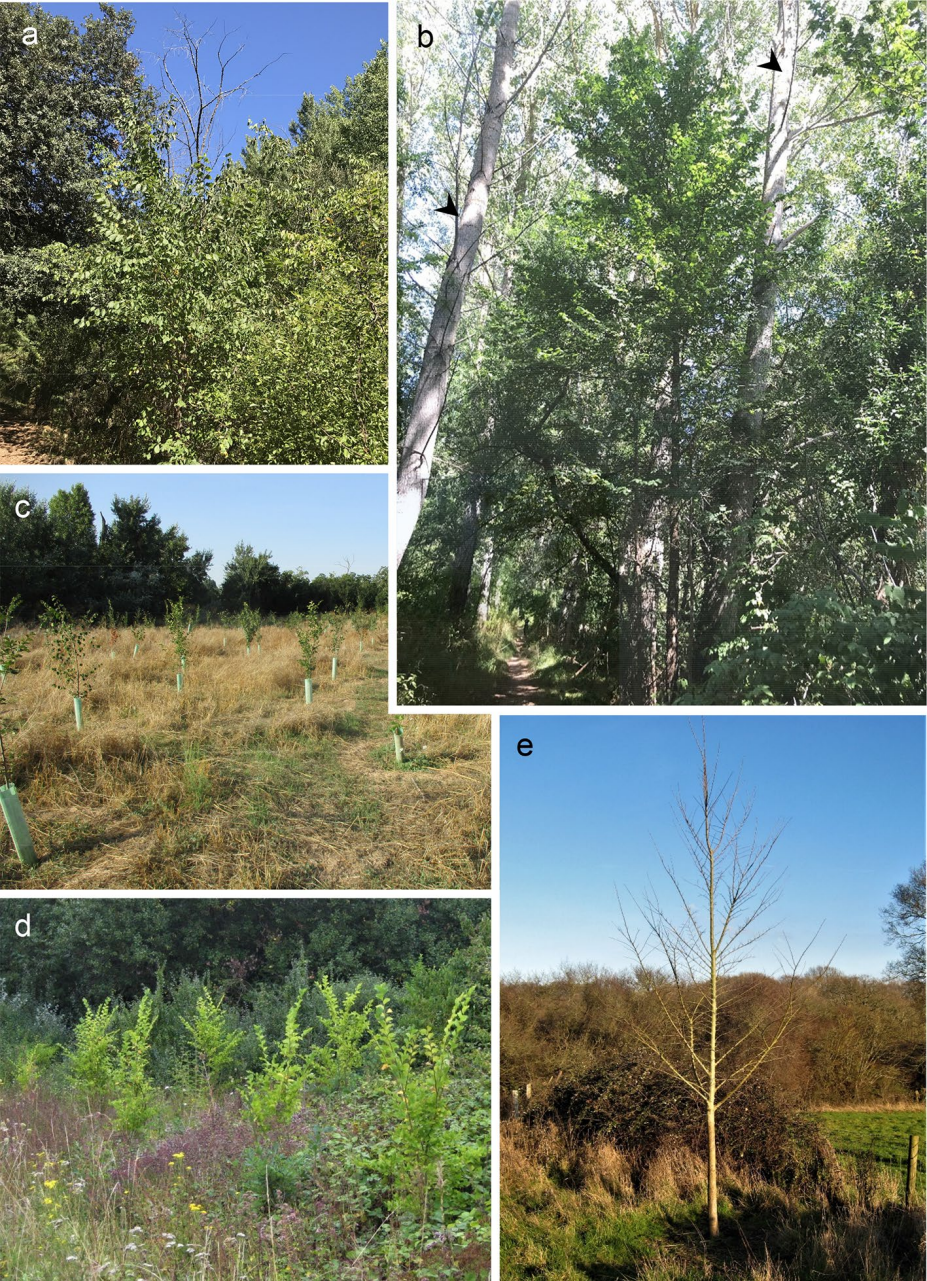
Natural and reintroduced *Ulmus minor* trees in Spain and the UK. **a** Vigorous elm sprouts in an open area of a riparian forest (Ebro River, Burgos, Spain) succumb to DED once they surpass approximately 5 m in height (see the dieback of the tallest stem). **b** Slender elm tree of circa 10 m in height growing in the same riparian forest, but in a closed canopy area dominated by *Populus alba* trees (arrowheads). In such situation, elms frequently show DED symptoms and rarely become dominant. **c** Young plantation of Spanish *U. minor* cultivars resistant to DED in Aranjuez, Madrid. **d** Plantation of the Spanish cultivar Ademuz, resistant to DED, in Portchester, UK. **e** Ademuz individual growing vigorously in Hampshire, UK. (Photos **d** and **e** kindly provided by Andrew Brookes, Butterfly Conservation)

Species within the genus *Ulmus* found in Europe and North America were formerly considered keystone species, but nowadays this status is severely degraded due to the global impacts of two fungal pathogens in the genus *Ophiostoma*: the causal agents of Dutch elm disease (DED). The first DED pandemic emerged in Europe in the first decades of the twentieth century and was caused by the introduction of *Ophiostoma ulmi*, which is moderately aggressive to European elms but highly aggressive to American elm (*Ulmus americana*) (Gibbs et al. 1975). This pandemic declined unexpectedly from ca 1935s in Europe, but not in North America (Peace 1960; Mitchell and Brasier 1994; Brasier and Webber 2019). Subsequently, a second, highly destructive pandemic caused by the introduction of *O. novo-ulmi* emerged in both continents. *O. novo-ulmi* proved to be highly aggressive towards both European and North American elms, and has now replaced *O. ulmi* in most locations (Brasier 2000a; Solla et al. 2008). The ability of some elm species to resprout vegetatively from the roots or reproduce abundantly by seeds has resulted in a post-epidemic phase in which recurrent cycles of high disease continue to occur in the recruitment trees once they reach 3–10 m in height (Brasier 1986a; Martín et al. 2019a; Brasier and Webber 2019) (Fig. 1a, b).

Since the pioneering work of Dutch workers in the 1930s (Heybroek 1957) many initiatives of elm reintroduction and restoration have been launched (Knight et al. 2017; Martín et al. 2019a) (Fig. 1c, d). The diversity of products and services derived from elms e.g., ecosystem protection, ornamental and landscape value, shade, timber and wood materials, biomass, nanomaterials (Heybroek 1993; Richens 1983; Martín-Sampedro et al. 2019; Mehmood 2019; Jiménez-López et al. 2020) are good reasons to promote the recovery of the elms. Current restoration initiatives use knowledge, techniques and plant materials developed by several different breeding programs and research groups (Smalley and Guries 1993; Mittempergher and Santini 2004; Pecori et al. 2017). Restoration of founder populations of elms should rely on two basic principles: availability of genetically diverse and arboriculturally suitable material resistant to DED (including native elm individuals) and deployment of appropriate planting strategies. Currently, both these requirements are far from optimal and elm restoration is still in its infancy.

In relation to plant material, availability of DED-resistant cultivars known to be suitably adapted for planting in natural habitats remains scarce, although in the next decade its availability is expected to increase (Pinchot et al. 2017; Martín et al. 2015, 2019a). A considerable number of hybrids obtained by interspecific crosses between susceptible native European elms and resistant Asian elms are available in the market (Santini et al. 2002, 2007, 2012; Buiteveld et al. 2015), but these are mainly used in urban forestry although their potential for timber or biomass production has been highlighted (Santini et al. 2010). However, because these genotypes are not native, their introduction in natural habitats can be controversial and often conflicts with social, ecological and political concerns. A more pragmatic and less ambitious alternative to the wide scale planting of DED-resistant cultivars is the deployment of diverse elm genotypes with partial resistance, in the knowledge that though many of them will suffer from the disease some may be in the right environment to survive and reproduce. This approach has been adopted by UK Conservation Foundation’s ‘Great British Elm Experiment’, and by several partners involved in elm restoration in France (Collin et al. 2020).

Planting strategies should also be derived from a deep knowledge of the ecology of the species and from experience of appropriate planting and aftercare methods. Because the use of elms in forest restoration was almost non-existent during the last century, current knowledge is limited. In spite of such limitations, pilot reintroduction plantings which deploy resistant ‘native’ *U. minor* cultivars in Europe (Martín et al. 2019a) and resistant *U. americana* cultivars in the USA (Knight et al. 2017), should enhance knowledge of elm biology and best planting practices. Common garden experiments (e.g. Martín et al. 2008a; Vivas et al. 2012; Martín et al. 2012; Griffin et al. 2017) and the establishment of experimental field plots used for research (e.g. Solla et al. 2015; Martín et al. 2021) could also enhance the experience needed for adequate elm restoration. Failures in attempts at elm restoration often arise from negative interactions between the planted cultivars and the environment e.g. ambient temperatures and soil moisture conditions. There is a need therefore, for breeders to identify negative interactions with the material before its release, and to properly inform foresters and arboriculturalists about any limitations on the performance of selected cultivars under certain conditions. For instance, the *U. minor* cultivar Christine Buisman, originally from Spain, was the first resistant cultivar released by the Dutch breeding program in 1937. However, it showed a poor adaptation to the Netherlands climate, being highly susceptible to coral-spot *Nectria* canker (Heybroek 1957).

**Fig. 2:**
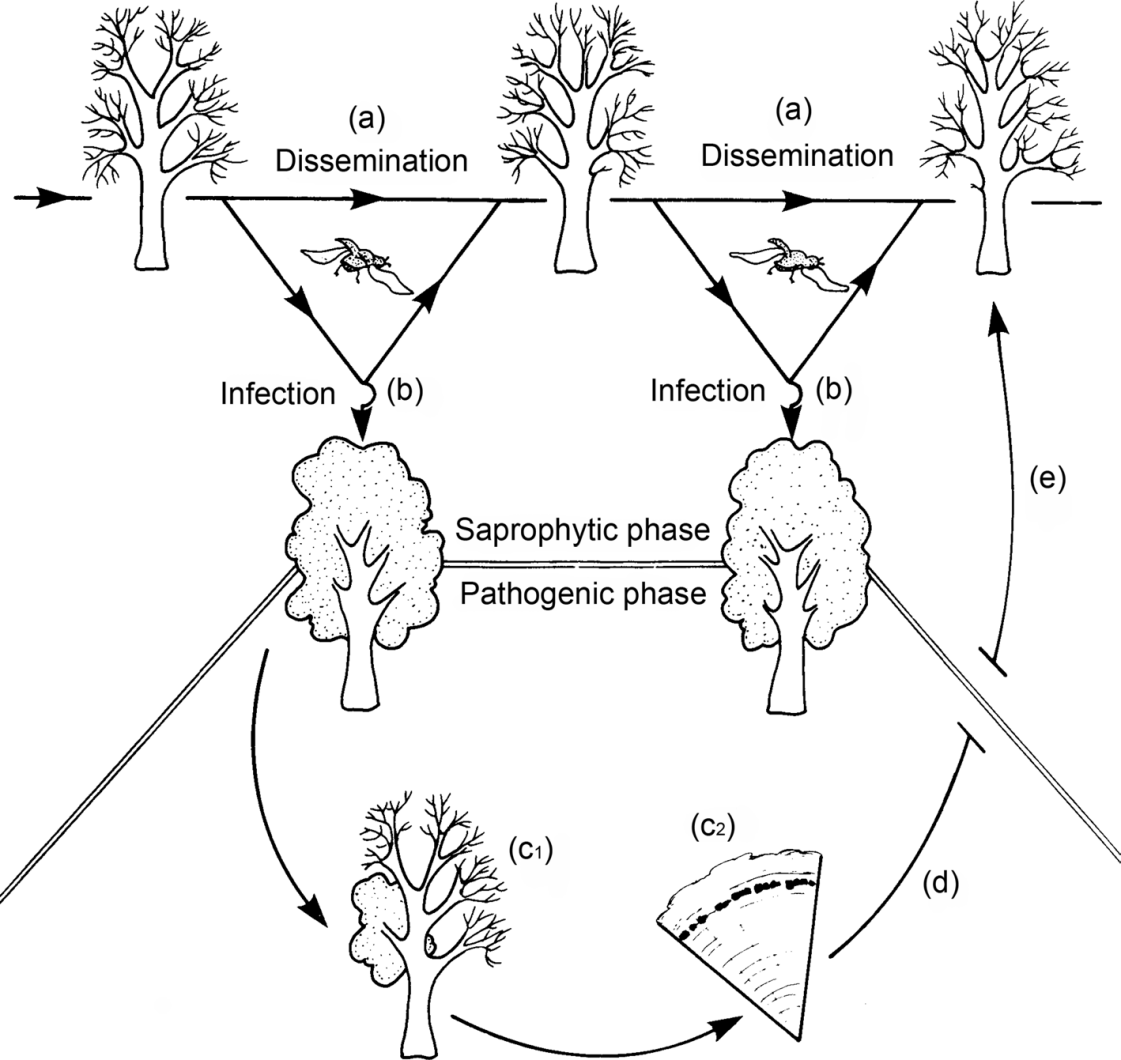
The two cycles of the pathogen in Dutch elm disease. **a** → **a** The continuous bark to bark cycle of the saprotrophic phase. **b** → **d** The pathogenic phase side loop, associated with beetle feeding and xylem infection. Adapted from Webber and Brasier (1984). **e** Studies with genetically marked isolates demonstrated that some genotypes from the pathogenic phase eventually feed back into the saprotrophic phase gene pool (Webber et al. 1988)

When resistance to DED is described here, it will refer to host resistance expressed in the xylem vessels after infection—the vascular wilt phase of the pathogen (Fig. 2; Webber and Brasier 1984). However, the pathogen has multiple ecological phases such that resistance to infection also occurs in the beetle feeding wounds prior to xylem infection, and this resistance may be a different process from that occurring in the xylem vessels (Webber and Brasier 1994). In addition, resistance will also occur in elm bark when the pathogen invades in association with scolytid elm bark beetles (Webber et al. 1988). DED resistance to the pathogen is variable among elm species and genotypes within species, and relies on constitutive and inducible defence mechanisms, with expression controlled by several genes (Townsend 2000; Aoun et al. 2010; Sherif et al. 2016; Beier and Blanchette 2018). Since the probable multigenic control processes involved in the resistance of elms to DED are poorly characterised and understood the potential use of genetic markers to accelerate selection for resistance, also remains limited. In this regard genome sequencing should be used in future to enhance breeding protocols through genome-enabled selection (Resende et al. 2012). This approach would use genomic information to predict the traits of interest and, consequently, reduce the time and cost of breeding. Genomic prediction models of DED resistance will, however, require complex studies involving large trials and accurate phenotyping of ‘resistance’. Attempts to produce DED resistant elms via genetic engineering, including the development of associated protocols of elm tissue culturing, induction of somaclonal variation and elm genetic transformation, were pioneered in the 1990s (e.g. Fenning et al. 1996; Gartland et al. 2000a,b, 2001) and may also play a significant role in the future.

The phenotype of a particular elm genotype is often strongly dependent on the environment where it develops and even on a particular season. The assessment of genotype × environment (G × E) interaction, i.e. the relative impact of different environmental changes on the phenotype (Isik and Kleinschmit 2003), should be determined in order to define the range of environmental conditions where a genotype would grow, reproduce and express resistance (Santini et al. 1997). The phenomenon of a genotype producing different phenotypes in response to different environmental conditions is known as phenotypic plasticity (Ghalambor et al. 2007). In DED, phenotypic plasticity of seasonal differences that occur in elms responding to infection has been described as a combination of host × environmental interactions prior to infection (host preconditioning), and fungal genotype × host genotype × environment interactions during and after infection (Brasier 1987). Although outstanding progress has been made in understanding plasticity of plant traits in response to abiotic factors, response to biotic factors has received considerably less attention (Solla et al. 2016; Valladares et al. 2007). Here we aim to explore the extent to which DED resistance is a plastic trait, the causes of this plasticity, and factors that could limit the negative impact of such plasticity in restoration programs. We will also consider how to avoid unintended consequences arising from the complexity of the ‘DED system’ when deploying DED resistant elms. For this purpose, early research on DED has been reviewed and contrasted with more recent research, with a particular focus on the experiences of the Spanish elm breeding program and UK studies on the pathogens and vectors.

## Phenotypic plasticity of foliage wilting of selected elms in response to DED

**Fig. 3:**
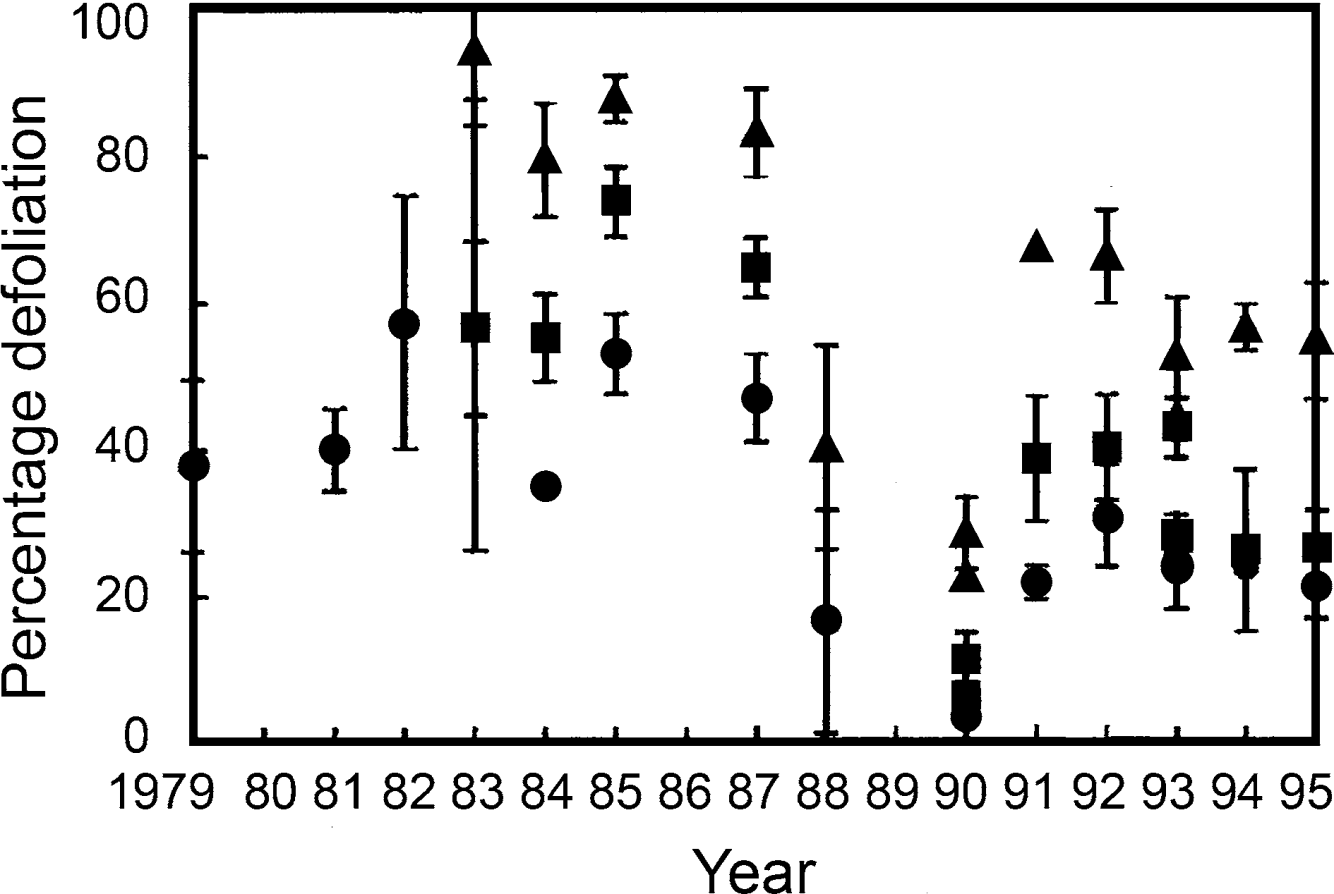
Levels of defoliation of *U. procera* (%) over eleven climatically variable seasons (1983–1995) caused by *O. novo-ulmi* SSNU isolates of high (filled triangle), medium (filled square) and exceptionally low (filled circle) aggressiveness (same isolates as in Fig. 7). Note the consistent order if the isolates across the tests. Bars, 95% confidence limits. Adapted from Sutherland et al. (1997)

In recent years, interest in planting DED resistant cultivars has been growing in Europe and North America. Given the scarcity of native resistant materials, these cultivars need to perform well under a variety of environmental conditions (Fig. 1c–e). Previous research on DED provides consistent evidence that elm resistance is highly plastic, and influenced by several climatic and edaphic factors (Kais et al. 1962; Sutherland et al. 1997; Solla and Gil 2002a). For example, during more than 11 years of elm inoculations at the same site using the single elm genotype *U. procera* SR4 (= *U. minor*), and the same set of *O. novo-ulmi* genotypes, the ranking of the pathogen genotypes remained the same but disease levels fluctuated from moderately resistant to highly susceptible, the main environmental influences being temperature and light intensity (Fig. 3; Sutherland et al. 1997). The genetic variation underlying such plasticity (i.e. in G × E interactions), however, has been less explored although it is likely to reflect a multigene resistance process. A major initiative to study DED resistance plasticity in the RESGEN 78 EU project (1997–2001), tested the performance of seven elm cultivars in common garden experiments across six European countries (Solla et al. 2005a). The cultivars showed different disease ratings depending on the location and the inoculation year, again indicating that DED resistance is a plastic trait. Although the susceptibility ranking of most cultivars again remained consistent regardless of the location and year, some significant G × E interactions were observed in the form of the inconsistent susceptibility of two of the elm clones in Germany and Italy. This result suggests that G × E interactions in elm resistance are not frequent, but that some cultivars can be remarkably sensitive to the environment.

**Fig. 4:**
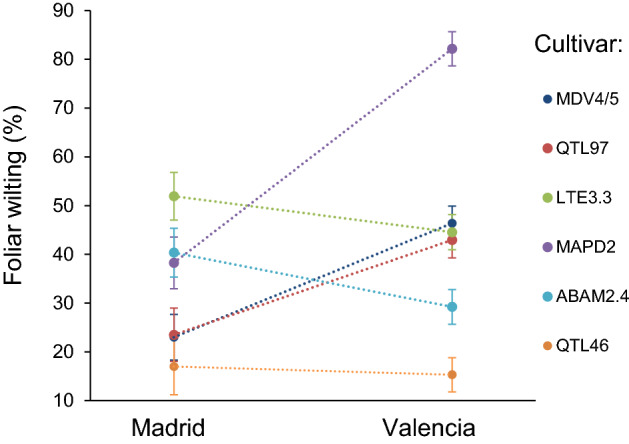
Interactions in leaf wilting symptoms at 60 days after *Ophiostoma novo-ulmi* inoculation between six *Ulmus minor* cultivars and two experimental locations (Madrid *vs* Valencia, Spain)

To explore phenotypic plasticity of DED resistance in Spain, common garden plots in contrasting environments were recently established. Although the research is still in progress, preliminary data confirms high plasticity of DED resistance and a significant genetic variation in such plasticity. For example, when comparing two experimental locations with contrasting climate, namely Madrid (inland Mediterranean climate; 700 m above the sea level (m.a.s.l.)) and Valencia (coastal Mediterranean climate; 40 m.a.s.l.), G × E interactions in resistance could be observed (Fig. 4). Thus, *U. minor* cultivar ABAM2.4 performed better in Valencia, in terms of resistance, in comparison to MAPD2, MDV4/5 and QTL97 which in contrast performed better in Madrid (Fig. 4).

Besides plasticity in DED resistance, other factors not always considered in elm breeding programs could also result in elm restoration failure. These include intolerance of selected cultivars to frost, flooding, wind and drought, and the relative attractiveness/susceptibility of selected cultivars to pests and pathogens such as scolytids, *Xanthogaleruca luteola*, *Cossus cossus*, *Stegophora ulmea*, *Nectria* canker and elm yellows (Smalley and Guries 1993; Pecori et al. 2017). Moreover, the presence of wild and domestic fauna in the plantation area can also reduce the survival of young trees.

## Unintended consequences derived from breeding: factors influencing symptom expression of selected elm cultivars

Resistant tree breeding is generally a long-term approach requiring sustained investment and a broad knowledge of the factors influencing symptom expression of trees. Loss of public confidence in the deployment of selected material can arise if resistance breaks down or resistant material is susceptible to other biotic or abiotic threats (Woodcock et al. 2019). The main objective of elm breeding is to deploy elms with durable, and therefore what is likely to be multigenic, or even multi-faceted, DED resistance. To this end, it is important to understand the resistance traits operating at different phases of the disease (Fig. 2). During the pathogenic phase in the xylem elms can display a variety of defence mechanisms, whose efficacy in pathogen suppression will lead to a ranking of resistance levels. Defence mechanisms can also be expressed during the colonisation of the inner bark (phloem) by the pathogen and beetle vectors, and during reinfection of elms in the beetle feeding grooves, and these too can translate into different levels of resistance. Furthermore, different elm species and genotypes can express different levels of attraction to the insect vectors. An ideal, though admittedly very ambitious, objective of elm breeding could be to integrate resistance mechanisms acting in different phases of the disease within the same elm genotype or population of genotypes. In the following sub-sections our understanding of the factors that influence resistance at different phases of the disease is reviewed.

### Xylem anatomy and phenology of wood formation

Secondary xylem of elms has a ring-porous structure. In spring, when water availability is not limiting, elms initially form wide earlywood vessels to optimize hydraulic efficiency, while in late spring and summer, when periods of water stress arise, they form narrow latewood vessels to increase hydraulic safety (Ellmore and Ewers 1985). The DED pathogens take advantage of xylem anatomy to spread efficiently through the wide earlywood conduits in the form of yeast-like blastospores, causing embolisms and inducing a wilt syndrome (Newbanks 1983). The pivotal role of the hydraulic conductive system in DED pathogenesis stimulated the search for anatomical characters involved in susceptibility. Several studies provided evidence that vessel diameter has a role in susceptibility to DED, as elms with narrow earlywood vessels tend to suffer less severe disease symptoms (Elgersma 1970; McNabb et al. 1970; Sinclair 1975; Martín et al. 2013a; Beier and Blanchette 2020). This conclusion is based on several assumptions. First, fungal propagules and toxins are likely to be more efficiently transported in wide conduits, which in turn are more prone to embolism than narrow vessels (Sperry and Tyree 1988; Pouzoulet et al. 2020). Second, the pathogen can be vertically compartmentalized (i.e. walled off) inside vessels if the lumen is blocked with tyloses and gums, but complete blocking of lumen is more difficult in wide vessels (Pouzoulet et al. 2020). Despite this theoretical framework, recent research has revealed that while susceptible *U. minor* trees tend to develop wide earlywood vessels, resistant trees do not always have narrow vessels (Martín et al. 2021). Instead, vessel occlusion by tyloses correlated with resistance only in a group of elms which had narrow earlywood vessels, supporting the hypothesis of easy pathogen compartmentalization in narrow *vs*. wide conduits.

Vessel size has been shown to be a plastic trait in different woody species, mainly influenced by variation in soil moisture content (Lovisolo and Schubert 1998; Solla and Gil 2002b; Martín et al. 2013a; Venegas-González et al. 2015; Noyer et al. 2017). In general, these studies indicate that plants under water stress tend to reduce vessel diameter. Such plasticity allows species to adapt to changing environmental conditions by supporting either hydraulic safety or hydraulic conductance, but in elms it can also potentially affect the DED susceptibility of certain cultivars by reducing susceptibility if environmental conditions induce the formation of narrow vessels. This is particularly relevant in screening tests for DED resistance because appropriate watering in spring and avoidance of stress factors (transplanting, pruning, wounding) is required prior to inoculation in order to facilitate pathogen dispersal within the tree (Tchernoff 1965).

**Fig. 5:**
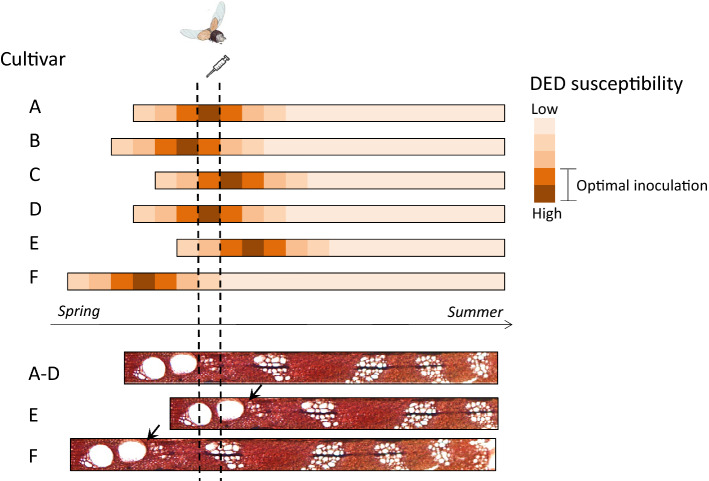
Diagrammatic representation of the susceptibility window of six elm cultivars to DED, with focus on insect vector flight period, inoculation date and phenology of wood formation. Optimal temporal window for artificial inoculation of cultivars **A–D** is represented within the two vertical dotted lines. This window often overlaps with the main flight period of adult elm bark beetles which transmit the DED pathogen during maturation feeding. Cultivars **A–D** are highly susceptible to DED as they have just formed wide earlywood vessels but have not developed latewood (see bottom panels representing xylem development). Cultivar E shows a late phenology; its earlywood vessels are still non-functional and, if inoculated, pathogen spread will be limited. Cultivar F shows an early phenology; by the time of the optimal inoculation window a proportion of latewood has already formed increasing hydraulic safety and decreasing the cultivars susceptibility to the pathogen. To properly characterize the resistance of cultivars E and F, a different inoculation date should be selected (black arrows)

The main period of DED susceptibility starts in spring, after earlywood vessels became fully functional, and lasts for 20–30 days, until a certain proportion of latewood is formed (Banfield 1941; Smalley 1963; Santini et al. 2005a), although this period varies markedly between elm genotypes (Smalley and Kais 1966). The natural synchrony between the peak elm susceptibility and spring flight of adult elm bark beetles which vector the disease (Fransen 1939) therefore contributes to the susceptibility of elms to DED (Solla et al. 2005b). Elm bark beetles in the genera *Scolytus* and *Hylurgopinus* transmit pathogen spores during maturation feeding in twigs and branches (Webber 2004; Anderbrant et al. 2017). If asynchrony occurs between DED vector transmission and earlywood vessel formation, disease symptoms are likely to be reduced, or absent (Fig. 5). For this reason, major northern and western range expansions of DED in Britain after the arrival of *O. novo-ulmi* coincided with hotter summers because of the influence on the flight period of the larger elm beetle, *S. scolytus* (Fairhurst and King 1983). Other research has found a significant correlation between DED resistance and precocity of flushing in Italian and French *U. minor* clones (Santini et al. 2005a). The authors argued that by time of pathogen inoculation, early flushing genotypes had already completed formation of earlywood vessels and were starting to lay down latewood, having passed the peak susceptibility window. Furthermore, they concluded that earlywood vessel formation is under strong genetic control (Ghelardini et al. 2006, 2010) and therefore could be a selectable trait in breeding programs. Highly significant differences in the timing of growth initiation were also observed among cultivars of *U. americana* but were not consistently associated with disease symptomatology (Townsend et al. 2005).

The timing of wood formation and its influence on DED resistance should therefore be taken into consideration during artificial screening for resistance (Beier et al. 2017a). The inoculation date has to be selected according to the tree phenology, which varies with latitude, climate type (e.g. maritime versus continental), and seasonal conditions (Ghelardini et al. 2006). In European elms, peak susceptibility is reached around 40–50 days after bud break (Tchernoff 1965; Solla and Gil 2003), and therefore monitoring of leaf phenology is strongly advisable, due to variability in phenology among cultivars. Otherwise, there is a risk of selecting ‘false’ resistant cultivars if susceptible trees are inoculated outside their susceptibility window (Fig. 5). To minimize this risk, it is advisable to characterise the phenology timelines of different cultivars before inoculation. If variation between cultivars is pronounced, inoculation should be carried out on different dates. Based on observations made in experimental plots established in Madrid (Spain), small differences in leaf phenology between *U. minor* cultivars were seen (around 5–10 days of variation in bud break among genotypes), but the differences were higher in Valencia (Spain) (around 5–20 days of variation) (unpublished results). While in Madrid it was possible to inoculate different cultivars in a single day, in Valencia cultivars had to be classified into two groups (early- and late-flushing trees) and inoculated on two different dates. Replication of inoculations across several years and locations is recommended to reduce the risks of false selection of resistant cultivars due to variability in phenology.

### The environment and the growth of trees

It is widely accepted that variation in climatic conditions can significantly alter the level of symptoms caused by DED (Kais et al. 1962; Smalley 1963; Tchernoff 1965; Sutherland et al. 1997). Climatic effects on host physiology, phenology and anatomy, among others, can influence the host response against infection and the ability of the pathogen to spread within the tree. Water shortage before infection has been shown to induce resistance to DED (Sutherland et al. 1997), while water stress after inoculation can enhance susceptibility (Solla and Gil 2002a). Temperature directly affects pathogen growth (Brasier et al. 1981) and the interactions between the host and the pathogen (Sutherland et al. 1997). These influences further highlight the importance of conducting resistance trials over several seasons and at different locations, as well as documenting as much as possible the experimental conditions under which the tests were carried out. Consideration should be also given to the climatic conditions during the process of inoculation. Conditions favouring a high transpiration rate such as hot and sunny weather, and the presence of available soil water will facilitate inoculum absorption and the development of external symptoms (Tchernoff 1965; Solla et al. 2001). For the same reason, inoculation should be preferably performed during the middle period of the day. Besides climate, other abiotic factors can also influence the level of resistance to DED. The presence of phenolic contaminants in soil and their absorption by roots has been shown to induce accumulation of suberin-like compounds in xylem tissues, leading to enhanced resistance to DED (Martín et al. 2008b, 2010b). This process was associated with the survival of *U. procera* trees in a stand in central Spain, where phenolic-based disinfectant products were frequently used for the cattle (Martín et al. 2010a).

**Fig. 6:**
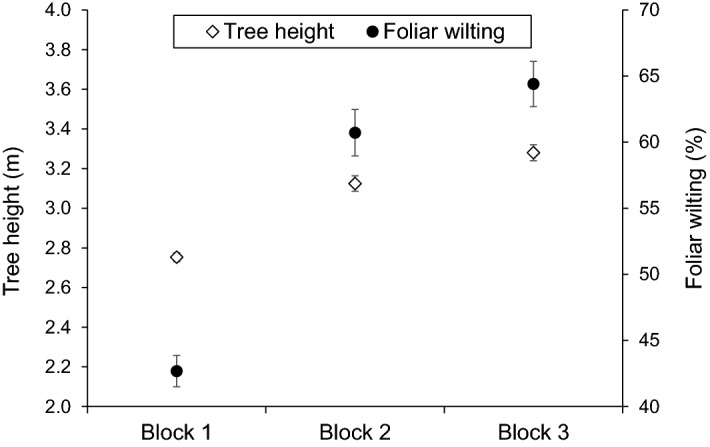
Mean tree height and leaf wilting symptoms shown by 160 *U. minor* progenies planted in an experimental plot at Puerta de Hierro Forest Breeding Center (Madrid, Spain). The plot included 2492 plants in an area of 1265 m^2^, and was divided in three blocks with a completely randomized design. Trees were inoculated with *O. novo-ulmi* in two consecutive years (2014 and 2015) at the age of 4 and 5 years. Tree height was measured the year before inoculation. Leaf wilting values correspond to 60 days after the second inoculation (vertical bars are standard errors). Note the low height of trees and the reduced symptoms in Block 1, as compared with Blocks 2 and 3. Block 1 had a background of more intensive cultivation, possibly resulting in nutrient deficiency

Any environmental factor which limits tree growth can potentially increase resistance to DED. Evidence from earlier studies (Heybroek 1957; Kais et al. 1962) and recent research on DED (Martín et al. 2021) indicates that vigorously growing elms tend to develop more severe foliar symptoms. In contrast, soils with a low water retention capacity, long-term flooded soils, infertile soils, or a prolonged dry season can reduce wilting symptoms (Sutherland et al. 1997). Screening tests for resistance need to ensure good physiological plant development before inoculation. Trees that were potted, wounded or placed under physiological stress in the previous year (e.g. by transplanting, pruning or girdling) should be avoided. In experimental plots it is also essential to include at least one susceptible cultivar as a control to check whether any stress factor is reducing plant growth and inducing resistance. Within the Spanish elm breeding program, inoculations performed in experimental plots with constrained tree growth resulted in low mean values for DED symptom expression. In addition, if soil properties or any other environmental factors are not uniform across the whole of an experimental plot, it should be divided into blocks to properly distinguish the environmental effects within the plot from host DED susceptibility (see Fig. 6). Despite this general trend, it is important to emphasise that the correlation between growth rate and DED susceptibility is far from absolute at the individual genotype level (Heybroek 1957), and thus resistant cultivars are not necessary always slow growers.

Under current climate change scenarios, where events such as prolonged droughts and flooding are increasingly frequent (IPCC 2014), it is important to characterize resistant cultivars and their potential to tolerate such events, and understand the influence of these events. Scientific data on disturbance tolerance in elms is scarce, and is mainly in regard to intraspecific variation. Optimal zones for planting different resistant cultivars, such as the distance from watercourses or oscillations in water table levels, need to be defined. To this end, pilot forest plantations can offer an ideal experimental framework to monitor the performance of elm cultivars. Such approaches can be further augmented by experiments to broaden our knowledge on elm ecophysiology and responses under different environmental constraints. For instance, Martínez-Arias et al. (2020) recently evaluated under controlled conditions the response of five resistant *U. minor* cultivars to waterlogging, to drought and to a combination of both stresses. They found that the five cultivars were similarly tolerant to drought, but the response to waterlogging and drought after a period of waterlogging significantly varied among genotypes.

### Sourcing the pathogen for elm screening

#### Ongoing evolution of the pathogen populations

In elm breeding, two key requirements are the selection of pathogen isolates for performing artificial inoculations and a good understanding of the pathogen and vector populations against which resistant elms are to be deployed. However DED is not caused by a single pathogen species, nor is it spread by a single species of vector, and the two introduced pathogen species, *O. ulmi* and *O. novo-ulmi*, have been in a highly unusual state of evolutionary flux because of extinction, hybridization events, and changes in selection pressures as the epidemics have progressed. Therefore it is desirable, when screening for resistance, to have an awareness of both past and current developments in a very dynamic and changing DED situation.

The importance of monitoring pathogen populations is emphasised by the arrival of *O. novo-ulmi* in the 1940s, which has resulted in the steady replacement and now near extinction of *O. ulmi* across Europe and North America. *O. ulmi* and *O. novo-ulmi* are differently adapted. *O. ulmi* is a relatively weak pathogen of European elms, has a slow growth rate at 20 °C but grows fast at  30 °C. *O. novo-ulmi* is aggressive on European elms, fast growing at 20 °C but slow growing at 30 °C (Brasier 1986a; 1991). Both species were probably introduced from East Asia. The first elm breeding programme, aimed at producing elms resistant to *O. ulmi,* was initiated by Dutch pathologists Christine Buisman and Johanna Westerdijk in the Netherlands in 1928 (Mittempergher and Santini 2004). However the programme was undermined by the discovery of a new ‘aggresssive strain’ of the pathogen, later designated *O. novo-ulmi* (Gibbs et al. 1972; Gibbs and Brasier 1973; Brasier 1991) and was re-orientated in the 1970s by screening the products against *O. novo-ulmi.* Because *O. ulmi* is a weaker pathogen and is now largely extinct in many locations it is no longer essential to use it in resistance screening. However, used in comparison with *O. novo-ulmi* it should still provide important clues to the genetic and physiological mechanisms of pathogenicity and resistance. It is therefore important that it is properly conserved in culture collections.

*O. novo-ulmi* itself is not a single entity but has spread as two subspecies: subsp. *americana* (SSAM) in North America and subsp. *novo-ulmi* (SSNU) in Europe and Central Asia. Among their multiple differences, SSAM is on average more aggressive to elms and faster growing than SSNU and has morphologically different perithecia (Brasier 1986b; Brasier and Kirk 1991). Furthermore, SSAM also exhibits a distinct substructure. The vegetative compatibility (vc) system (self –non-self recognition system) of *O. novo-ulmi* is central to the organisation of its populations (Brasier 1986a). Surveys across North America have shown that two near clonal vc and molecular lineages, designated AMSG and EUSG, comprise > 60% of the SSAM population, alongside a genetically highly heterogeneous component including probable SSAM × EUSG recombinants (Brasier 1996; Brasier & Kirk 2000). No differences in growth rate or pathogenicity were found between SSAM, EUSG or the heterogeneous component. In the 1960–70s elements of this SSAM population were introduced to western Europe, where a Spanish survey demonstrated two divergent molecular groups within SSAM, designated Ona Groups 1 and 2 (Solla et al. 2008). A recent genome-wide study of the DED pathogens has also identified two genetically distinct lineages within SSAM, labelled AME1 and AME2 (Hessenauer et al. 2020). Isolates in common to the studies indicate Ona 1 and AME1 correspond to the EUSG and its closer genetic derivatives, and Ona 2 and AME2 to the AMSG and its derivatives. Isolates belonging to AME1 exhibit faster mycelial growth in vitro and higher divergence in genes overexpressed during the yeast phase (Nigg and Bernier 2016) compared to AME2 isolates (Hessenauer et al. 2020).

Whether migrating as SSAM or SSNU, *O. novo-ulmi* has evolved rapidly through hybridization, introgression and selection since the 1940s (Brasier 2001; Brasier et al. 2004). In this, *O. ulmi* initially played a critical role. During its replacement by *O. novo-ulmi* transient, largely unfit hybrids were formed (Kile and Brasier 1990; Brasier et al. 1998) via which *O. novo-ulmi* acquired *O. ulmi* loci including the *MAT-1* mating type locus, vc (*vic*) loci (Paoletti et al. 2006), pathogenicity loci (Et-Touil et al. 1999) and temperature response loci (Brasier et al. 1998; Et-Touil et al. 2019; Hessenauer et al. 2020). As a result *O. novo-ulmi* populations migrating across Europe changed rapidly from being largely clonal to highly genetically heterogeneous in just a few years, including Portugal and Spain (Brasier 1988; Brasier and Kirk 1991; Brasier et al. 2004). Many genes acquired from *O. ulmi* were soon lost, but the *O. ulmi MAT-1* and *vic* loci became fixed in *O. novo-ulmi* populations (under selection pressure from deleterious RNA viruses) and *O. novo-ulmi* now carries *O. ulmi MAT-1* and *vic* loci almost by definition (Brasier 2001). In North America a similar but much slower introgression-driven evolution of *O. novo-ulmi* SSAM has also occurred. This has included the long term survival of the dominant AMSG and EUSG near-clones. Their continued dominance could be due to the high susceptibility of *U. americana*, low virus pressure, their competitiveness in the saprotrophic phase, and the low diversity of *O. ulmi* in North America (Brasier and Kirk 2000).

Furthermore, as a result of the geographical overlap of the two *O. novo-ulmi* subspecies SSAM and SSNU in Europe since the 1970s, swarms of SSAM × SSNU hybrids have occurred freely in the overlap zones (Brasier 2001; Santini et al. 2005b; Solla et al. 2008; Brasier and Kirk 2010; Hessenauer et al. 2020). By the 1980s  more than 70% of the *O. novo-ulmi* population at sites in the Netherlands and Italy were SSAM × SSNU hybrids. The hybrids exhibit novel *O. novo-ulmi* phenotypes, and as a result of selection, locally adapted genotypes may be emerging (Brasier et al 2021). *O. novo-ulmi* is, once again, reinventing itself in Europe.

The complexity and dynamics of the above events again emphasises the need for continued monitoring of changes in the pathogen populations across Europe, Central Asia and North America.

#### Selection of isolates for resistance screening

**Fig. 7:**
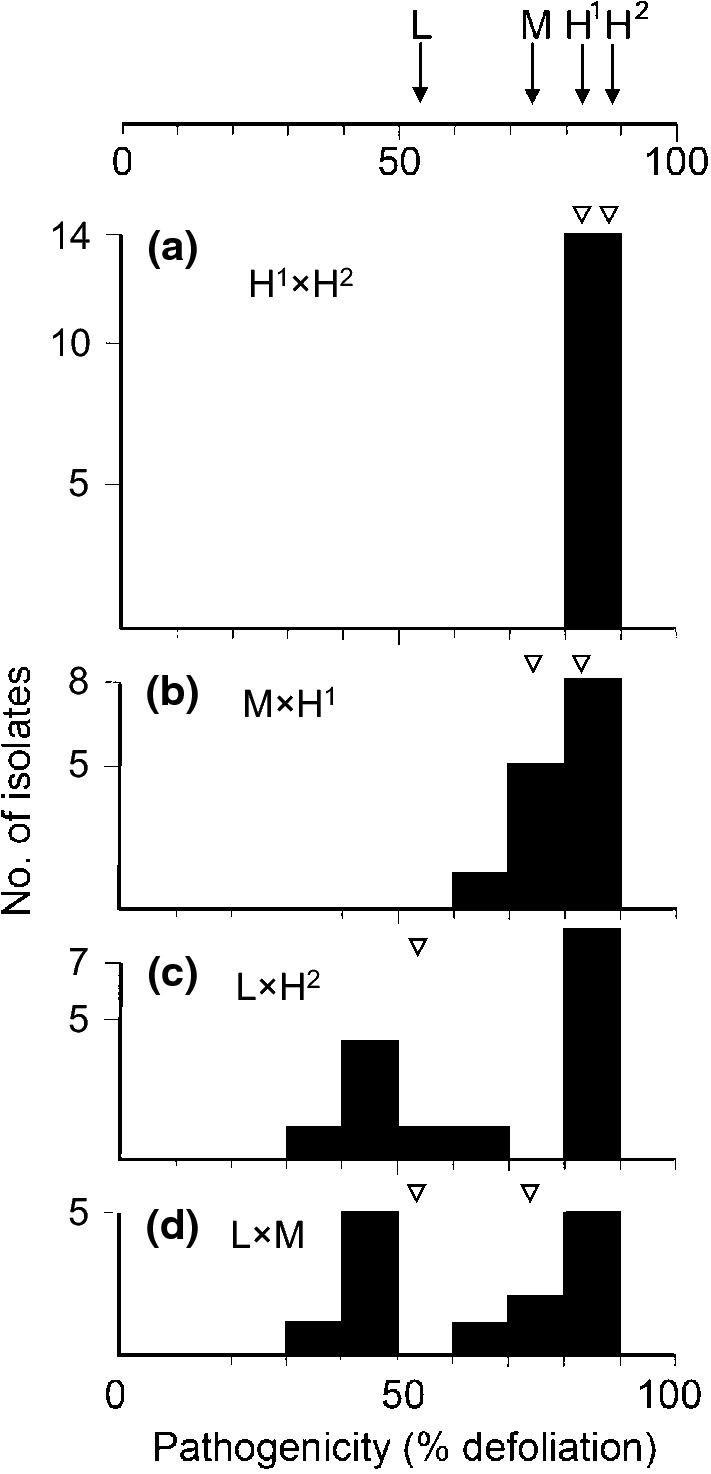
The heritability of pathogenic aggressiveness in *O. novo-ulmi* SSNU. Top line, the % defoliation of clonal *U. procera* caused by isolates of typically high, (H), typically medium (M), and exceptionally low (L) aggressiveness. **a**–**d** Pathogenicity distributions of progenies from crosses. Crosses **a** H^1^ × H^2^ and **b** M × H^1^ show largely additive variation in aggressiveness. Crosses **c** and **d**, between H^2^ and M and L, the isolate with unusually low aggressiveness, show segregation of a single gene. The isolate involved (AST27), from an epidemic front site in Iran, was subsequently shown to be carrying a single introgressed *O. ulmi* pathogenicity gene (Et-Touil et al. 1999). Adapted from Brasier (1987)

Against this background, what isolates should be used for resistance screening? It is scientifically desirable, but not essential, to understand what form of *O. novo-ulmi* is present in the vicinity in which the material will be released. In North America this is likely to be ‘pure’ SSAM, although this population is nonetheless intrinsically variable as confirmed by the occurrence of two genetic lineages in this subspecies (Brasier and Kirk 2000; Solla et al. 2008; Hessenauer et al. 2020). In eastern Europe and central Asia it is likely to be ‘pure’ SSNU, also variable (Brasier 1986b). In Western Europe there is an increasingly complicated SSAM × SSNU hybrid situation (cf. Brasier and Kirk 2010). Nonetheless several lines of evidence argue for simply using the most aggressive *O. novo-ulmi* genotypes available locally for screening. First, even in ‘pure SSNU’ populations some highly pathogenic genotypes of SSNU are present, an example being SSNU isolate H327 widely used as a standard in pathogenicity comparisons (cf. Brasier 1987; Et-Touil et al. 1999). Second, within *O. novo-ulmi* (SSAM or SSNU) inheritance of aggressiveness appears to be largely additive (multiple genes of small effect). Thus there was no evidence from crosses between moderately aggressive (M) and highly aggressive (H) *O. novo-ulmi* isolates for the segregation of major genes in the pathogen (Fig. 7a, b; Brasier 1987). An exception were the progenies of high × low and medium × low aggressiveness crosses, which segregated 1:1 (Fig. 7 c, d; Brasier 1987). This was later shown to be due to an introgressed *O. ulmi* pathogenicity gene in the low aggressiveness isolate (Et-Touil et al. 1999).

Third, as already mentioned, when *O. novo-ulmi* isolates of statistically different levels of aggressiveness are inoculated into the same *U. procera* clone across many seasons, they typically retain the same ranking order in terms of % defoliation (Fig. 3), another indication that the pathogen × host interaction involves multiple, largely additive pathogenicity genes. Fourth, there is evidence that in the current post-epidemic period (with often very large numbers of small recruitment elms, infection levels periodically high and (in Europe) often smaller less effective vectors predominating) the most aggressive pathogen genotypes are being favoured, probably as a result of directional selection for higher aggressiveness (Brasier and Webber 2019). Also, the SSAM × SSNU hybrids now emerging widely in Europe are significantly more pathogenic than their SSNU parent and just as highly pathogenic as SSAM, again probably due to directional selection (Brasier and Kirk 2010; Brasier et al 2021). Taking all the above factors into account, *O. novo-ulmi* isolates of suitably high aggressiveness are likely to be available for screening in most locations.

A related question is during what phase of the DED cycle is it best to collect isolates for elm screening: from beetle feeding wounds, infected xylem, or the bark (or phloem) of diseased elms? There are in fact two pathogen cycles in DED (Webber and Brasier 1984). The pathogen’s saprotrophic phase—or ‘bark to bark phase’—when it lives in and around the beetle breeding galleries in dying elm bark (Fig. 2a), is the main gene pool of the pathogen. The beetles carry spores of the pathogen from the bark to bark continually across the years, and it is during this phase that the pathogen is at its most active and abundant: sexual recombination takes place, a turnover of genotypes occurs (via competitive growth and vegetative incompatibility interactions), and RNA viruses spread between the mycelia (Brasier 1986c; Webber et al. 1988). For this reason, the saprotrophic phase is best avoided when collecting isolates solely for screening.

**Fig. 8:**
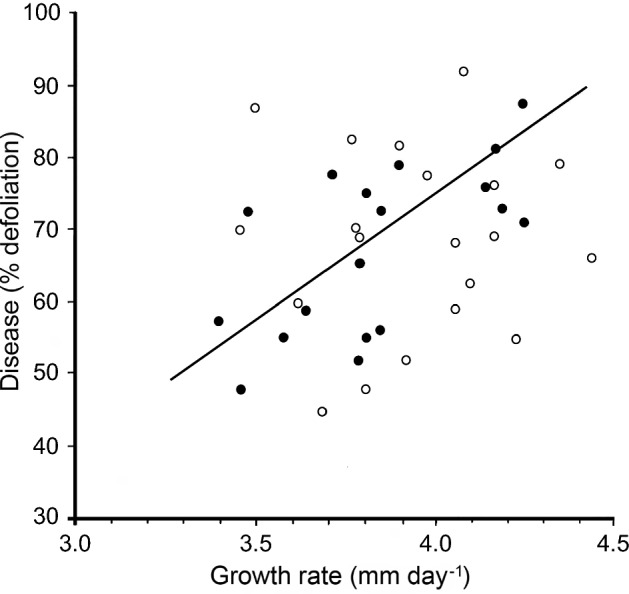
Pathogenicity versus growth relationships of *O. novo-ulmi* SSAM isolates sampled from the pathogenic phase in xylem (open circle) and the saprotrophic phase in the bark around beetle galleries (filled circle) at the same disease site in Britain. Only the pathogenic phase isolates showed a correlation between these two key fitness parameters (r = 0.63). These isolates would have been subject to rigorous selection in the feeding wound and in the xylem. Adapted from Brasier and Webber (1987)

The other pathogen cycle in DED is the pathogenic phase (Fig. 2b–d), which begins when the pathogen grows in a feeding wound and enters the xylem, causing the foliar wilt. This phase is in reality just a side loop from the bark to bark cycle. Nonetheless, it is also the phase where strong selection for pathogenic ability, including ability to colonise the feeding wound and ability to spread in the xylem, will occur; and the phase in which deleterious virus infections are probably lost. Experiments have shown that the putatively fitter genotypes from the pathogenic phase can pass back into the main gene pool in the bark: a genetic feedback loop (Webber et al. 1988). Essentially the pathogenic phase is a genetic sieve. The potential difference between the pathogenic and saprotrophic phase gene pools can be seen in Fig. 8, which compares pathogenic and saprotrophic phase isolates collected from the same disease site (Brasier and Webber 1987). Only the pathogenic phase isolates showed a clear correlation between growth rate and pathogenicity. The saprotrophic phase isolates did not: they were altogether more variable. Therefore, because pathogenic phase isolates are on average likely to be fitter and more aggressive, due to host selection, collecting cultures for screening from the xylem of trees with advanced wilt symptoms is recommended.

Note that it is probably the feedback loop from the pathogenic phase to the saprotrophic phase (Fig. 2) that facilitates any directional selection for *O. novo-ulmi* aggressiveness in the current, post epidemic period. This raises the issue of the likely impact on *O. novo-ulmi* populations of any deployment of resistant elms. When deployed in small numbers (relative to nearby susceptible elms) they will probably exert only limited selection on a local *O. novo-ulmi* population. Were they to be deployed in large numbers and some local *O. novo-ulmi* genotypes infected the xylem and caused dieback, it is theoretically possible that a gradual increase in pathogen aggressiveness might occur via the feedback loop. On present evidence, however, this appears unlikely. First it appears that in the current post epidemic period *O. novo-ulmi* may already be close to its maximum pathogenic aggressiveness (Brasier and Webber 2019; Brasier et al 2021). Second, any further increase in pathogenic fitness will probably be set against the need to maintain growth, reproductive and general competitive fitness in the saprotrophic phase and then successfully re-infest the next generation of emerging beetles (Webber and Brasier 1984; Brasier 1986a).

**Fig. 9:**
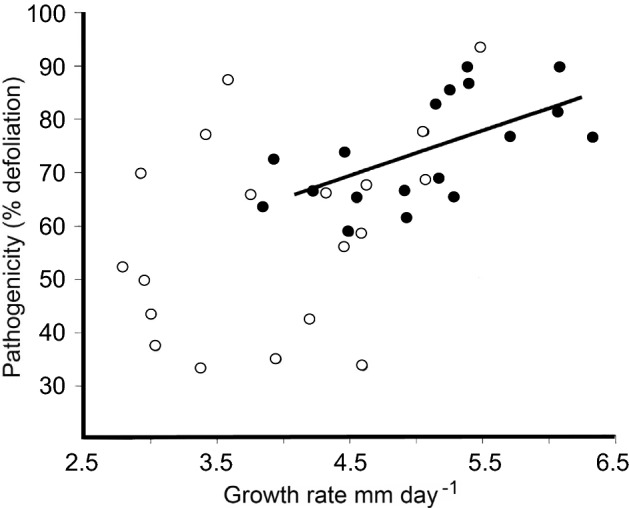
Pathogenicity versus growth relationships of *O. novo-ulmi* SSAM isolates of different mating types, MAT-1 (open circle) and MAT-2 (filled circle) from the early epidemic stage in Britain. Only the MAT-2 isolates showed a correlation between the two fitness parameters (r = 0.57). Adapted from Brasier and Webber (1987). Note that the MAT-1 isolates were on average slower growing and less pathogenic than the MAT-2 types. This is probably because some of them carried not only a recently introgressed *O. ulmi* MAT-1 locus but some other introgressed *O. ulmi* genes (Brasier 2001); and because MAT-1 s tend to be specialised for sexual reproduction (Brasier 1986a)

Even the sexual compatibility type (or mating type) of an *O. novo-ulmi* isolate can influence its pathogenicity. On average, *MAT-1* types are slightly less pathogenic (and slower growing) than *MAT-2* types on *U. procera*, but this difference is not detectable in all tests due to seasonal variations in host susceptibility. Also some *MAT-1* isolates, including H327 (see above), are just as pathogenic as *MAT-2* s. This trend is probably due to *MAT-1* s tending to be significantly more fecund than *MAT-2* s (Brasier 1986a) and therefore more specialised in sexual reproduction: a division of labour. In one test (Fig. 9) an extreme difference between pathogenicity of *MAT-1* s and *MAT-2* s was detected among wild isolates and progeny of *MAT-1* × *MAT-2* crosses (Brasier and Webber 1987). In this case only the *MAT-2* s showed a correlation between pathogenicity and growth rate. The isolates came from the early epidemic period when *O. ulmi* was in decline. The result was probably due to the *MAT-1* isolates having arisen de novo via introgression of the *MAT-1* locus from *O. ulmi*; and to their still carrying other *O. ulmi* genes of relatively strong and negative effect on their pathogenicity and growth. In most present day, European, post-epidemic *O. novo-ulmi* populations the more ‘negative’ *O. ulmi* genes have probably been eliminated by selection. Therefore it may be less necessary now, in the post epidemic period, to know the mating type of an *O. novo-ulmi* isolate used in resistance screening. Nonetheless it is always valuable to have as much scientific information on the pathogen genotypes used as on the host.

Often, probably only a single genotype infects the xylem from a beetle feeding wound. However there have been suggestions to use mixtures of pathogen isolates in elm screening. This may sometimes reflect a degree of uncertainty about the quality of the inoculum. For instance, in the frame of the Italian elm breeding program a mixture of one isolate per each of the two *O. novo-ulmi* subspecies was used to challenge candidate clones (Santini et al. 2008). Elm trials in North America have sometimes used a mixture of *O. ulmi* and *O. novo-ulmi* isolates (Townsend et al. 1995; Smalley and Guries 1993; Pinchot et al. 2017), possibly because the high susceptibility of *U. americana* results in smaller differences in aggressiveness between the two species*.* However an argument can be made that *O. ulmi* + *O. novo-ulmi* species mixtures should be avoided because of the possibility of induced resistance to *O. ulmi* (Scheffer et al. 1980; Solla and Gil 2001a; Hubbes 2004), especially in moderately resistant European elms, although some studies have found little effective response (Sutherland et al. 1995). Mixtures of *O. novo-ulmi* genotypes are likely to compete numerically and perhaps qualitatively as budding yeasts in the xylem vessels. They may also compete in growth rate, and via antagonistic vegetative compatibility reactions, when growing as hyphae across vessel end walls. Mixtures of *MAT-1* and *MAT-2* mating types should probably also be avoided because they are programmed to interact sexually. Therefore without strong experimental evidence for a greater efficacy of isolate mixtures in screening, including over multiple seasons, and why, they are probably best avoided. Statistically sound scientific data on the behaviour of elm varieties and genotypes may best come from inoculating them with a single, well characterised pathogen genotype (e.g., Martín et al. 2015).

Finally, assessment of pathogen aggressiveness should preferably be undertaken by inoculating elms older than 3 years (Solla et al. 2005c), although early screening tests using young plantlets have been also developed (Green et al. 1985; Martín et al. 2019b).

#### Handling and conservation of pathogen isolates

**Fig. 10:**
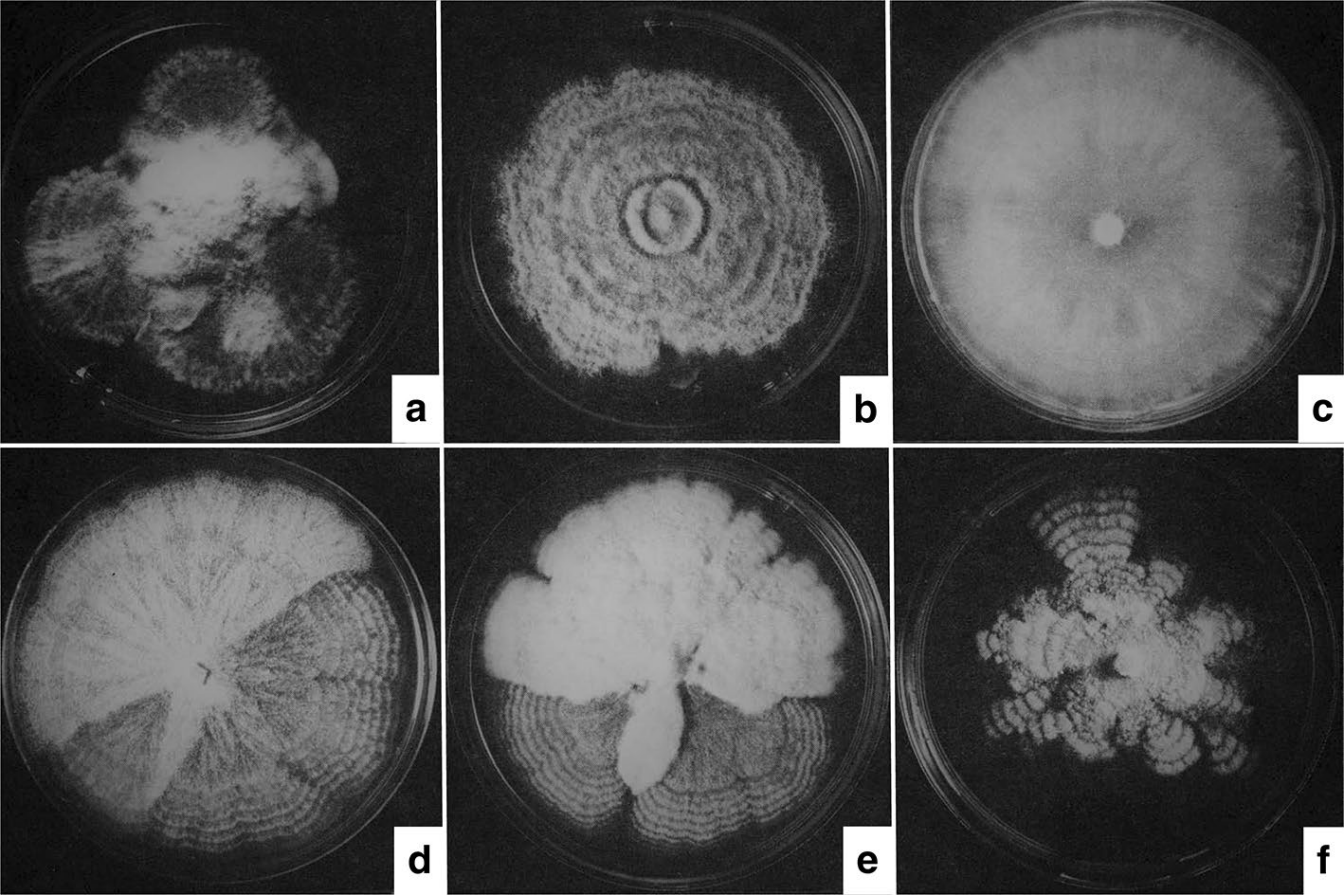
Examples of degenerate cultures of the pathogens. **a** A culture of *O. novo-ulmi* SSAM severely degenerated after storage under mineral oil. **b**, **c** Common degenerate forms of *O. ulmi*. **d** An *O. novo-ulmi* SSAM wild-type colony (lower part) giving rise to a more densely mycelial non wild-type sector (above). **e** An example of a commonly seen dense, felty non wild-type sector (upper part; in this case arising in a rare *O.novo-ulmi* isolate carrying introgressed *O. ulmi* genes). **f**, The unstable 'amoeboid' colony type that occurs naturally with severely virus-infected isolates of *O. novo-ulmi*. The cultures shown in a-e are all laboratory artifacts and are not normally found among fresh wild isolates. Adapted from Brasier (1982)

While the use of pathogen reference isolates with demonstrated high aggressiveness in previous screening tests is recommended (Santini et al. 2005a; Buiteveld et al. 2015) it should also be noted that without careful handling isolates of both *O. novo-ulmi* and *O. ulmi* easily degenerate in artificial culture, probably due to stress effects. This is usually manifested as sectoring and/or a change from the original wild type pattern to a variety of more densely-mycelial, sometimes pigmented, non-wild type forms (Fig. 10). Cultures in institutional culture collections, unless stored under very specific conditions (e.g. stored at − 80 °C, a reasonable guarantee of genetic stability), are often degenerate and unsuitable for study, including altered pathogenicity to elm. Many were probably degenerate before being stored. Some authors working on *O. ulmi* in the 1930–60 s were unwittingly describing degenerate isolates. The same applied to *O. novo-ulmi* in the 1970s. If the same isolate is to be used for screening over several years, therefore, taking sub-cultures from altered sectors of growth, or from cultures that are old or drying out, should be avoided. Rather than risking this problem it may be better to collect fresh local cultures annually during late winter or early spring from xylem of elms (whether naturally infected or inoculated) that were heavily diseased in the previous season. Alternatively the original wild type colony pattern of an isolate should be noted (perhaps recorded photographically) and probably reconfirmed annually. During screening, as well as the selected isolate, other isolates of known aggressiveness can be tested as controls, ideally using a moderately susceptible host. For methods of isolation and culture maintenance see e.g. Brasier (1981).

### Pathogen inoculation protocols, and the implications of vector feeding behaviour, for elm screening

Methods used to inoculate elms with the pathogen include making pinpricks in the young shoots high in the crown (H.S. McNabb method, Iowa State); making small cuts with a scalpel in ~ 2 year old twigs no more than one fifth of the way down the crown’s height (e.g. Tchernoff 1965, method 6; Gibbs et al. 1972); making a 4-mm-deep hole one-half m from the ground and injecting a spore suspension into the hole (Beier et al. 2017b); inoculating bark cores 8–10 cm above soil level (e.g., Solla and Gil 2002b); and inoculating root flares via a 0.5 inch borehole (e.g. Pinchot et al. 2017). The pinprick method aims to more closely mimic the mode of pathogen entry via a beetle feeding wound, and the ‘one fifth down the crown’ method somewhat the same, the rationale being that when an infection is initiated via a feeding wound the pathogen has to spread downwards against the sap-stream from the infection site. Inoculation near the base of a tree is probably more equivalent to the transmission that occurs via root grafts between adjacent diseased and healthy trees. In nature, this can lead to a more rapid and extensive collapse of the crown. As there is probably no one ideal method, when testing promising resistant elm material an argument can be made to use both (1) a more delicate ‘within the high crown’ inoculation to mimic natural field exposure of the material; and (2) a more robust basal inoculation test that should put the material under the strongest possible pathogen pressure.

In this regard, the numbers of spores used to infect elms during screening is usually of the order of ~ 500,000 (ie. 5 × 10^5^). This figure is at the high end of the numbers reported to be carried by scolytid beetles. The species of *Scolytus* involved in disease transmission vary in size, carry different average spore loads of the pathogen, and tend to breed in elms of different bark thickness (Webber and Brasier 1984; Webber 2004). They therefore vary in their likelihood of initiating an infection. They also vary in their relative frequency during and after an epidemic, and therefore in their potential to influence current disease levels. A method for estimating the numbers of spores required for xylem infection, using artificial ‘beetles’ and creating wounds in twig crotches similar to feeding wounds, showed that at ambient humidity around 1000 spores were needed to infect *U. procera* (Webber 1987, 2004). Xylem infection frequency at 1000 spores was around 20%, rising to 40% with 50,000 spores.

In parallel studies the number of spores carried by the larger European elm bark beetle *S. scolytus* under UK conditions averaged 50,000, with an upper level of ~ 700,000; whereas those carried by the smaller European elm bark beetle *S. multistriatus* averaged < 200 spores with an upper level of 200,000 (Webber and Brasier 1984). *S. multistriatus* emerging from breeding material in Spain in summer carried a similar number of spores, but the much smaller *S. kirshii* from the same elm material almost no spores (Webber 1990). However the number of spores carried will also vary according to the position of the pupal chambers, bark moisture, bark thickness, and the season of emergence (Webber and Brasier 1984; Faccoli and Battisti 1997). Thus in Italy over half of the individuals of *S. multistriatus* and of the very small *S. pygmaeus* emerging in spring carried the pathogen, whereas in summer less than 10% of the beetles did so (Faccoli and Battisti 1997).

Broadly DED is all about the interaction of the host, pathogen and vector populations: it is a multiple host-multiple pathogen and fungal virus-multiple vector system in which critical thresholds often lead to either explosive or quiescent disease (Webber and Brasier 1984). In the current post epidemic period in Europe, with large numbers of mainly small elms under cyclical attack, *S. scolytus* is at a disadvantage due to a shortage of suitable sized breeding material and *S. multistriatus* and other smaller vectors are more significant in disease transmission (Brasier and Webber 2019). Further, in the absence of *S. scolytus*, *S. multistriatus* and smaller vectors may sometimes have access to thicker bark for breeding than they had during the main epidemic, and so carry more spores. If large elms of moderate resistance begin to dominate the landscape again in future a resurgence of *S. scolytus* is likely. Whether this might also occur if large numbers of highly resistant elms were deployed is unclear, but an increase in the *S. scolytus* population would probably accelerate any evolution, via the feedback loop (Fig. 2), towards increased aggressiveness in *O. novo-ulmi*.

Considering that resistant elm material needs to be useful for tens if not hundreds of years, by concentrating breeding and selection almost entirely on resistance to the pathogen in the xylem we may be overlooking other useable aspects of host resistance, including beetle feeding preference and resistance in the beetle feeding wound. Thus the ‘main’ European vector, *S. scolytus*, shows host species feeding preferences. It preferentially feeds on *U. procera* or *U. minor* rather than either *U. glabra* or *U. laevis* (Webber and Kirby 1983; Webber 2000; Sachetti et al. 1990), yet both *U. glabra and U. laevis* are more susceptible than *U. procera* on inoculation (Brasier 1977).

Resistance to the pathogen is not confined to the xylem. Differential resistance to the pathogen has been demonstrated in the bark (phloem) of feeding wounds. When artificial feeding wounds in *U. pumila* were infected with 10,000 *O. novo ulmi* spores 70% resulted in xylem infection, but without any external symptoms (Webber and Brasier 1994). On the moderately resistant Commelin elm (a complex *U. minor* × *U. glabra* hybrid; Heybroek 1993) 10,000 spores resulted in only 30% xylem infection, though some external symptoms developed. In *U. procera* the xylem infection levels were ~ 70% i.e. similar to *U. pumila*, and external symptoms were always observed. The minimum spore thresholds resulting in infection of *U. pumila,* Commelin and *U. procera* were 500, 1000 and 500 spores respectively. Apparently the immunity of *U. pumila* to DED does not preclude successful entry by the pathogen. It also appears that Commelin elm may have an active resistance mechanism in the bark around the feeding groove (and maybe in the bark elsewhere in the tree). The possibility that this resistance is derived from its *U. glabra* parentage needs investigation (Webber and Brasier 1994). Combining resistance to beetle feeding and the infection process with xylem resistance in elms might considerably enhance their field performance.

It should be noted that the feeding wound is also the stage during which the pathogens’ RNA viruses or d-factors have most effect. By reducing spore viability and growth rate of *O. novo-ulmi* (and *O. ulmi*) in the feeding wound the more deleterious viruses can significantly reduce or prevent xylem infection (Brasier 1983; 1986a; Webber 1987; 1993; Sutherland and Brasier 1997). By exerting a degree of biological control, they may have contributed to the unexpected decline of the first DED pandemic in Europe (Brasier and Webber 2019). Early in the second epidemic in Europe the viruses spread readily in the *O. novo-ulmi* clones at epidemic fronts and might have supressed this epidemic if *O. novo-ulmi* had not acquired the *MAT-1* an*d vic* genes from *O. ulmi,* increasing its resistance the viruses (Brasier 2000b). The existence of what appears to be a single clone of *O. novo-ulmi* SSAM in New Zealand still offers an opportunity for biological control of the pathogen by deploying viruses (Brasier 2000b).

## Introgression of genes from susceptible and exotic elms into resistant cultivars

When resistant elm cultivars are introduced into the field, uncertainty may exist about their long-term performance and that of any outcrossed progeny produced later. If the cultivars have outstanding DED resistance and low phenotypic plasticity for this trait, the short-term impact of DED in the planting area should be low or negligible. As resistance to DED has a high level of additive genetic control (Solla et al. 2015), the progeny of fertilisation within the planted cultivars can also be expected to show a high level of resistance. However, there is also the possibility of out-crossing between the resistant cultivars and local, surviving susceptible elms. This risk is enhanced by the dispersal distances of elm pollen (up to 8 km in *U. minor*) (Bertolasi et al. 2015). Although in Europe most susceptible mature elms have been killed by DED, abundant small elms still survive as seedlings, sprouts and root suckers (Brasier 1986a; Nielsen and Kjær 2010; Brasier and Webber 2019). In years of low disease incidence, these flowering, small surviving elms will sustain gene flow between isolated trees and stands. In some cases, mature elm trees also survive in certain locations (Bertolasi et al. 2015; Martín et al. 2006). For instance, a considerable number of large individuals of the Atinian elm (*U. procera*) still survive in Spain despite the high susceptibility of this cultivar to *O. novo-ulmi*, possibly due to environmental resistance (Martín et al. 2006). Since Roman times, this cultivar was massively propagated and planted in the Iberian Peninsula and England, and served as living support of the grapevines (Gil et al. 2004), among other uses. The genotypic diversity of field elm in these regions was dominated by this single cultivar, which could have had consequences for the fast pathogen spread in epidemic fronts (Martín et al. 2010c). The survival of susceptible trees implies that the long-term resistance of the progeny of resistant cultivars may be reduced where susceptible elms occur in sympatry with the introduced population. Indeed, recent research has shown that in resistant × susceptible progenies DED resistance can be overcome by recurrent infections (Martín et al. 2021).

**Fig. 11:**
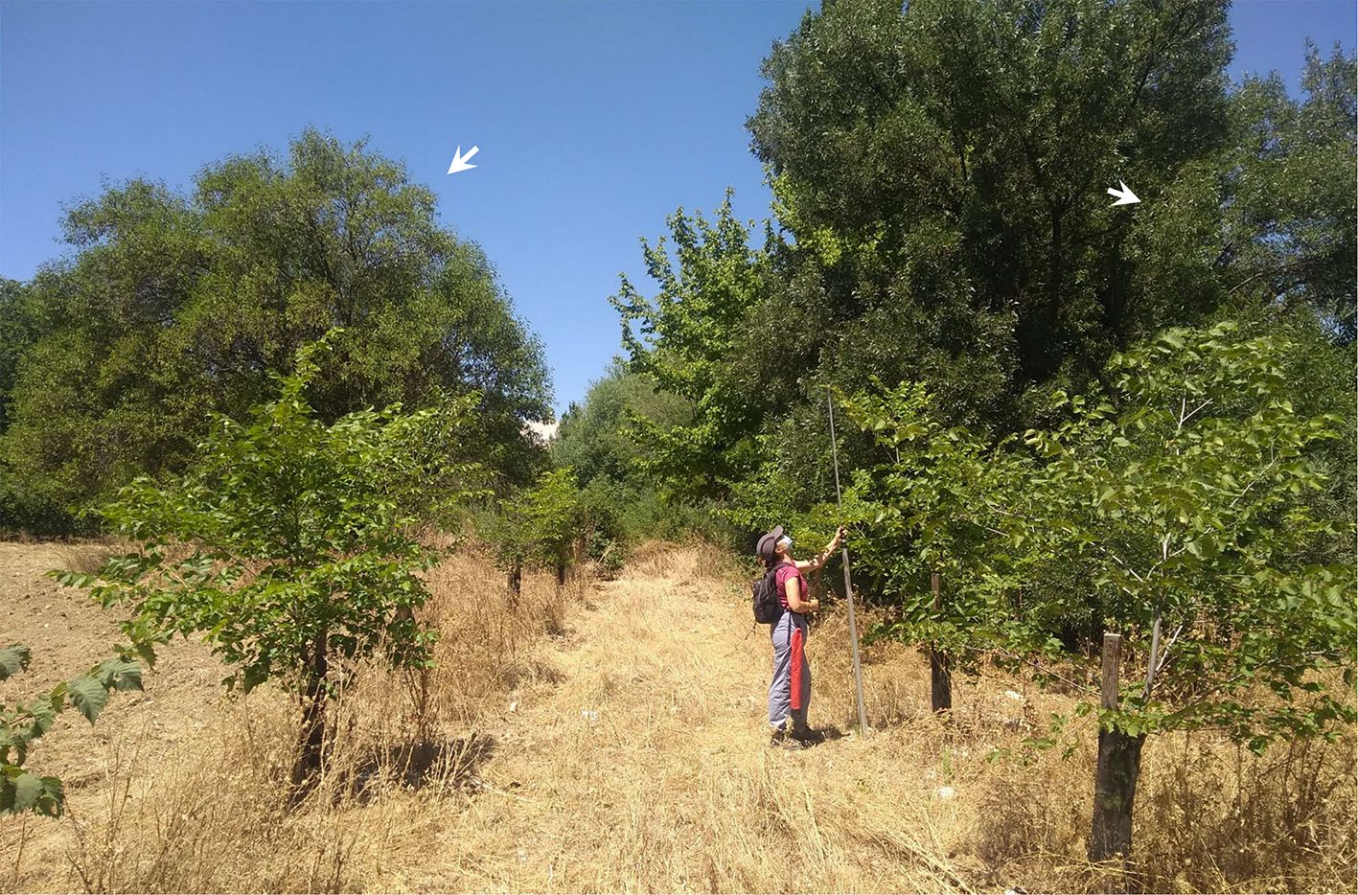
Plantation of native *Ulmus minor* trees on a plain area of the Meaques stream (Madrid, Spain). The presence of exotic Siberian elms near the plantation (arrows) will probably threaten the native germplasm of future elm generations in the area

Another concern for resistant material is the risk of genetic introgression from introduced exotic species or from hybrids (Fig. 11). This risk is particularly high with regard to the spread of the Siberian elm (*U. pumila*), which was introduced into Spain in the sixteenth century (Cogolludo-Agustín et al. 2000) and in the USA from about 1900 (Zalapa et al. 2009) as an ornamental, and into Italy in the 1930s, at the time of the first DED epidemic, to replace the lost elms, which, were widely used in agriculture (Brunet et al. 2013). In all these countries *U. pumila* has shown high reproductive potential, and a tendency to high hybridization rates, traits that may increase the potential for invasiveness (Ellstrand and Schierenbeck 2000). Indeed, it has been shown that Siberian elm readily hybridizes with *U. minor* in Europe (Cogolludo-Agustín et al. 2000; Brunet et al. 2013) and with *U. rubra* in the USA (Zalapa et al. 2009; Brunet et al. 2016), forming large hybrid populations. Siberian elm has, in general, moderate to high resistance to DED (Smalley and Guries 1993), and has therefore been used in several breeding programs as a source of resistance genes through backcrosses. However, conservation concerns in Spain have led to a prohibition of the marketing of *U. pumila* and its hybrids for use as a forest tree.

The ease of hybridization between *U. pumila* and *U. minor* led to questions about the native character of seven resistant *U. minor* cultivars selected by the Spanish elm breeding programme (Martín et al. 2015). Selected cultivars were identified based on their morphological characters as *U. minor*, but in some cases distinguishing any hybrids from native *U. minor* by morphology alone is very difficult. Preliminary results through single nucleotide polymorphism (SNP) marker analysis indicate that two out of the seven resistant elms may carry *U. pumila* genes (unpublished results). These two clones (Toledo and Fuente Umbría) have been provisionally withdrawn for use in natural forest habitats. Any new, putatively resistant *U. minor* cultivars are now being analysed with SNP markers to characterize their genetic background.

There are contrasting opinions about the deployment of hybrids or introgressants in natural areas and on their potential impact in elm reintroduction in the longer term. It could be argued that given that *U. pumila* and hybrids are already widespread in the landscape, resistant native cultivars used in restoration will inevitably outcross with *U. pumila* or elm hybrids in future, and that preventing such hybridization is not technically feasible. Such an argument is however, in contradiction to the aim of restoring the native species, given the potential invasiveness of the hybrids. From an ecological perspective, *U. pumila* is less tolerant of flooding than European elms (Heybroek 1979), and therefore introgression with *U. pumila* genes could reduce the average fitness of offspring of reintroduced *U. minor* in riparian areas. In this case, natural selection may favour the persistence of genotypes with native traits. Further, a positive outcome of such introgression could be a higher resistance to DED in the progeny (Solla et al. 2015; Pecori et al. 2017), or better adaptation to aridification resulting from climate change, since *U. pumila* is well adapted to semi-arid conditions (Heybroek 1979). Independently of these possibilities, the selection of planting areas should take into account the presence of exotic or susceptible elms in the area and the potential impact on the planted elms.

## Changes in the host microbiome

In recent decades, it has become increasingly apparent that the response of plants to external stress factors is strongly influenced by their symbiotic microbiome. The microbiome structure presents an additional component to be considered in plant breeding programs (Wei and Jousset 2017). In particular, microorganisms inhabiting the rhizosphere, phyllosphere and the internal tissues of leaves, roots and stems (endophytes) have been shown to play a role in plant resistance against biotic and abiotic stress (Redman et al. 2011; Raghavendra and Newcombe 2013; Witzell et al. 2014; Busby et al. 2016; Terhonen et al. 2019). Although the underlying mechanisms are far from understood, variation in host microbiomes across environments may contribute to phenotypic plasticity in resistance traits.

A bark endophyte of *U. glabra*, the ascomycete *Phomopsis oblonga*, significantly lowers the transmission rate of DED by competing with and limiting the breeding and development of elm bark beetles in *U. glabra* (Webber 1981; Dvořák et al. 2012). Some bacterial and fungal isolates artificially inoculated into the xylem of elm trees have also been found to enhance DED resistance (Lam et al. 1987; Bernier et al. 1996; Scheffer et al. 2008; Martínez-Arias et al. 2021). However, whether the natural host microbiome contributes to DED resistance under field conditions remains poorly understood. In recent years, the Spanish elm breeding programme has initiated a study of the potential associations between DED resistance and composition of the fungal endophyte flora in stem tissues. Using isolation and culturing the frequency and diversity of fungal endophytes was compared between 10 elm genotypes of varying resistance level to DED (Martín et al. 2013b). The study revealed that resistant clones tended to harbour a lower frequency and diversity of xylem endophytes compared to susceptible trees. Therefore, the possibility that use of resistant elms in forest restoration might have an unintended effect on the diversity of fungi colonizing xylem tissues deserves further research. Subsequently, a metabarcoding study on the same elm genotypes detected around twelve times more endophytic fungal taxa (213 OTUs) than the culture-based approach. No relationship between DED resistance and fungal diversity estimates in stem tissues (xylem plus internal bark tissues) was detected. However, the relative abundance of two orders of endophytic fungal yeasts (Chaetothyriales and Cystobasidiales) was correlated with the resistance of elm genotypes to DED (Macaya-Sanz et al. 2020). Functional traits of these yeasts are currently being investigated and, in preliminary tests, competitive mechanisms with *O. novo-ulmi* were observed (Martínez-Arias et al. 2021). The yeasts also improved root growth through production of IAA (Martínez-Arias et al.; unpublished results). This may be a mechanism through which the tree vigour is improved and survival of abiotic stress is enhanced. Indeed, although these observations were host genotype-dependent, they suggest that endophytic yeasts might influence plant homeostasis during stress, and therefore reinforce the defence response of resistant genotypes during infection.

The interaction of the local environment with the tree microbiome could be another factor underlying the variation in resistance to DED. Indeed, the stem fungal endobiome in *U. minor* appears to be strongly influenced by geographic location (Macaya-Sanz et al. 2020). Equally, however, a small proportion of the fungal endobiome appears ubiquitous across all environments (Macaya-Sanz et al. 2020). This highly stable core endobiome may have important functional roles in the ecophysiology of the host (Shade and Handelsman 2012).

Clearly the role of the elm microbiome needs further research. In particular, its possible contribution to DED resistance and the possibility that it could be managed to increase tolerance to DED in restoration plantations. Manipulation of the host microbiome would probably be more effective during the early developmental stages of seeds or seedlings, due to the priority effects that occur in endophytic microbial assemblages (Tosi et al. 2020). The effects of any such manipulation would need to be evaluated long term, not only with regard to DED resistance, but also to plant survival and growth in different environments.

## Other factors likely to affect plantations of resistant elms

Unexpected mortality of elm trees may occur following mechanical coring with an increment borer. Six out of eight mature *U. minor* trees in Rivas-Vaciamadrid, Spain (Martín et al. 2006) died from internal spread of *O. novo-ulmi* the year after being cored, while adjacent non-cored trees remained asymptomatic (unpublished results). Following natural infection and recovery of the trees several years previously, the borer wound probably resulted in colonization and collapse of the current functional rings, allowing the pathogen to escape from previous radial compartmentalization. The literature indicates that risk of hardwood mortality caused by increment coring is low (Helcoski et al. 2019), but reports usually consider wounds as entries for fungi that accelerate wood decay, not as pathways of colonization when a vascular pathogen is inside a tree.

The emergence from neighbouring trees of beetles carrying spores of the pathogen early in the season, when moister bark may favour sporulation (Webber and Brasier 1984; Faccoli and Battisti, 1997), could be more unfavourable for newly established trees. If infection is combined with phloem girdling by the beetles and/or drought, young planted trees could fail. For this reason it is often recommended that young trees are planted at a lower density in zones at high risk from the pest or the pathogen (Woodcock et al. 2019). Mass trapping has been suggested as a method for reducing populations of *Scolytus multistriatus* (O’Callaghan and Fairhurst 1983; Paine et al. 1984), but is not recommended in plantations because of the risk of attracting beetles carrying the pathogen. The bark of elms under stress from causes other than DED is also attractive to breeding beetles (Baker and Norris 1968). Indeed, pruning wounds made on various species of healthy elms, including *U. procera* and *U. americana*, can significantly increase the number of beetles attracted to the trees (Byers et al. 1980; Landwehr et al. 1981). Severe pruning should therefore be avoided, particularly, during the temporal window of elm bark beetles flight (e.g., from April to October in Madrid, Spain; Solla et al. 2005b). Summer drought can also increase beetle pressure and contribute to tree failure, while high temperatures, and high light intensity and drought can supress foliar symptoms (cf. Sutherland et al 1997). During two hot dry years in southern Britain in 1975–6, at the local peak of the second epidemic, some avenues of mature elms showed no crown symptoms but, under resulting climate stress, were subject to girdling as a result of mass breeding attacks by *S. scolytus.* They also showed heavy xylem streaking due to the resulting colonisation by *O. novo-ulmi*.

In an elm plantation, several other circumstances may benefit the survival of trees. If a low pathogenic *O. novo-ulmi* strain (or *O. ulmi*) infects a moderately resistant tree and a certain threshold of foliage wilting is not exceeded, this tree will probably recover and will be less susceptible to further infections by *O. novo-ulmi* (Solla and Gil 2001b; Hubbes 2004). Furthermore, if sequential infection occur (i.e. in consecutive years), elm trees will have less chances of recovery compared with longer time periods between first and further infections (3 or more years; Solla and Gil 2001b).

There are still substantial gaps in our knowledge of the processes that regulate the beetle-fungus symbiosis as well as the vector-host tree relationship. The association between elm bark beetles and DED fungi depends on many factors, such as climatic and other environmental variables and the interactions between the different components of the biotic community. For example, many other organisms such as mites may play a role in the fertilisation and spread of the pathogen (Brasier 1978; Moser et al. 2010) while other fungi, bacteria and viruses can reduce the pathogen’s survival potential through nutrient competition or parasitism (Webber and Hedger 1986; Webber 1993, Brasier 2000b; Pepori et al. 2018). Similarly, parasitism by fungi and nematodes influences the survival of the vectors. Such factors may yet have a role in the restoration of elms in the landscape.

Chemical communication is of potentially critical importance to the management of vector populations. Pheromone traps are already used to attract and detect the elm scolytids and it is known that chemicals attract the vectors to each other for aggregation and probably to the host for maturation feeding and breeding (e.g. Lanier et al. 1976, 1977; Grove 1983; Klimetzek and Kopp 1983). Chemical signals are also involved in the beetle feeding preferences. For instance, twig bark extracts from *U. laevis* and *U. glabra* induce less *S. scolytus* feeding than extracts from *U. minor* and *U. pumila* (Pajares et al. 2004). Similarly, bark extracts from certain *U. minor* genotypes induce less feeding activity than extracts from other genotypes of the same species (Pajares et al. 2004). Also, infection by *O. novo-ulmi* induces chemical changes in the elm that attract vectors to the trees for breeding (McLeod et al. 2005). The identification of any chemical compounds that make individual trees more attractive to beetles is of primary importance and could provide another avenue of marker-assisted breeding for native elm species (Pajares 2004; and *cf*. Büchel et al. 2016). Ultimately, integration of several, if not many different methods of DED control may be the most effective way forward.

## Unintended biosecurity breaches

Epidemics caused by introductions of alien pests and pathogens are increasing in frequency as a result of growth in the international movement of plants (Brasier 2008; Santini et al. 2013). Good biosecurity is therefore important when moving elm material between centres, countries or between continents. Fortunately, being scolytid vectored, the DED pathogens are unlikely to be spread by movement of living elm plants, though their international movement via diseased elm logs or bark is well known (cf. Brasier and Gibbs 1973). Nonetheless it is likely that *O. novo-ulmi* SSNU was introduced to Uzbekistan (central Asia) in the 1970s by elm breeders working in Tashkent Botanic Garden, who inoculated elms with cultures brought from Volgograd (Russia). Probably only *O. ulmi* was present previously in central Asia.

The transport or introduction of living elm cuttings or saplings is, however, a much greater risk because of other non-native pests and pathogens they may harbour. In Britain alone, the elm yellows mycoplasma, the elm zig-zag sawfly and the apple root knot nematode (origin probably Japan) have all been introduced or detected in the past decade, and there is evidence that the nematode (EPPO 2017; Prior et al. 2019) and the mycoplasma were introduced on young rooted plants of resistant elms. The nematode is believed to have been imported to the Netherlands on elms from Japan for resistance breeding prior to World War II, and was subsequently distributed to at least ten other European countries on elm selections (EPPO 2017). The mycoplasma- infected stock in the UK was destroyed and the pathogen probably eradicated, but the sawfly and the nematode were already beyond effective control. Beyond this, there are other DED pathogens in Asia, including *O. himal-ulmi* on *U. wallichiana* in the Himalayas (Brasier and Mehrotra 1995) and another on *Zelkova* in Japan (Masuya and Brasier, in preparation) that could pose a threat to Eurasian and North American elms in future. It is therefore essential that strict biosecurity procedures are adopted, including if possible a year or more of quarantine and expert observation in the receiving country, when moving elm material across borders.

## Conclusions

Complex environmental, biological and genetic factors can influence the behaviour of elm trees exposed to the DED pathogens and to their beetle vectors, potentially leading to uncertain or unintended outcomes during elm resistance testing and elm reintroductions. To avoid unwanted outcomes and to enhance long term elm durability in the context of changing pathogen, vector and disease pressures, a range of current and future actions are recommended:

- To evaluate the optimal geographical and climatic range of promising elm cultivars, tests for resistance to the pathogen should be undertaken over several years, and under contrasting climate and soil conditions (i.e. tests for host × pathogen × environment interactions). Ideally, tests should include abundant replication of target elms, and some more susceptible cultivars as controls.
- Resistant elm cultivars with a relatively uniform response to the pathogen across different environments should be deployed in locations where a wide range of environmental conditions are likely to be encountered. Cultivars with a high plasticity are likely to be useful only in locations which allow optimal resistance traits to be expressed.
- *O. novo-ulmi* isolates for use in artificial inoculations to test for resistance are probably best obtained locally, from xylem of heavily diseased trees. Any isolates older than 1 year should be checked to ensure no degeneration in culture has occurred. If possible the subspecies and lineage status, mating type and the comparative aggressiveness of tester isolates should be determined. With promising elm selections both high canopy and basal stem inoculation methods should be considered.
- On promising cultivars, in addition to xylem resistance, the potential for resistance to pathogen infection via beetle feeding wounds could also be investigated before release, using artificial feeding wounds (see Webber 1987). If possible alongside control cultivars of known feeding groove resistance (e.g. *U.* × *holandica* ‘Commelin’ and *U. procera*).
- Similarly, the relative attractiveness of a cultivar to beetle feeding could also be investigated before release, again in comparison with species/cultivars of known (low or high) attractiveness (e.g. *U. glabra* and *U. procera*).
- A combination of xylem resistance, feeding wound resistance and low feeding attraction could considerably enhance longer term field resistance of cultivars and their commercial and environmental utility.
- Ideally the influence of local disease pressures (size and density of susceptible hosts, pathogenic variability of the pathogen, beetle vector species) around selected, substantial populations of released elms could be monitored experimentally to assess the risk of directional selection for increased aggressiveness in *O. novo-ulmi*.
- In geographic locations where elm populations have frequent seed set, progenies of resistant selections should be monitored after deployment for any acquisition of genes (via introgressive hybridization) from native susceptible or exotic resistant elms.
- DED susceptibility in *U. minor* is directly correlated with geographic origin and date of bud burst, early flushing clones showing the least symptoms. This suggests that earliness of bud burst represents a mechanism of disease avoidance owing to an asynchrony between the susceptible period in the host and the time of natural infection by bark beetles. If precocity can be shown to be under genetic control it could also be selected for in a breeding programme.
- The symbiotic microbiome of selected cultivars might play a significant role in DED resistance. If confirmed, the microbiome of elm selections could be monitored or even manipulated to ensure that they are favourable to the maintenance of resistance.
- High standards of biosecurity including quarantine should be employed when moving resistant elms or other elm material between biogeographic zones or internationally.
- In future biotechnology should enhance our understanding of the various resistance processes in elms and therefore the potential to deploy trees with highly durable and stable resistance in elm restoration. However because of the complexity of the DED pathosystem it remains uncertain whether elm genotyping (genome sequencing) will provide additional resistance markers suitable for accelerating the selection process.
- As resistant elms need to be deployed for many decades, if not centuries, elm breeding programmes cannot afford to get into the host–pathogen arms races, often involving rapid loss of resistance and a need for continued deployment of new genotypes, that characterise some agricultural host–pathogen systems (cf. Fry 2008; Croll and Lane 2016) and lead to the rapid loss of resistance and need for continued deployment of new genotypes. The need for highly durable resistance in elm implies aiming to achieve multigenically controlled, largely additive or quantitative resistance, avoiding dependence on major genes.

## Data Availability

The datasets generated during and/or analysed during the current study are available from the corresponding author on reasonable request.
